# Diversification of West Nile virus in a subtropical region

**DOI:** 10.1186/1743-422X-6-106

**Published:** 2009-07-16

**Authors:** Daniel M Chisenhall, Christopher N Mores

**Affiliations:** 1 Louisiana State University, School of Veterinary Medicine, Department of Pathobiological Sciences, Skip Bertman Dr., Baton Rouge, LA 70803, USA

## Abstract

**Background:**

West Nile virus (WNV) has spread across North, Central, and South America since its introduction in 1999. At the start of this spread, Florida was considered a potentially important area with regards to transmission due to its geographic, climatological, and demographic conditions. Curiously, the anticipated high levels of transmission or disease outbreaks have not been observed. As other studies have predicted that the lack of intense WNV transmission is not due to vector incompetence, we sought to evaluate the role of viral strain diversity in WNV transmission in Florida. Therefore, a phylogentic analysis was carried out on several isolates collected from three distinct locations in Florida.

**Results:**

Contrasting with a positive control collected in Indian River County, Florida during 2003 that contains the original NY99 genotype with valanine at amino acid 159 of the envelope region, all of the isolates collected in 2005 contain the WN02 genotype composed of a substation with alanine at that position indicating the window of introduction of the WN02 genotype occurred between 2003 and 2005. From the eight isolates collected in Duval, Indian River, and Manatee Counties; there is also a silent nucleotide substitution that differentiates the isolates collected on the Atlantic side of the state compared to the isolate collected on the Gulf side, which groups closer to isolates from other locations near the Gulf.

**Conclusion:**

As a whole, the Florida isolates contained numerous variable nucleotide and amino acid sites from the reference sequences, as well as each other; indicating greater nucleotide diversity within the Florida 2005 isolates than within other regions. Finally, a series of three amino acid substitutions surrounding a set of histidines located in the envelope coding region that hypothesized to play a role in conformational changes was found in the isolate collected in Indian River County, perhaps changing the antigenicity of the homodimer. Taken together, these findings expand our understanding of the temporal and spatial compartmentalization of West Nile virus subtypes within North America.

## Background

West Nile virus (WNV) is a member of the family *Flaviviridae *and in particular, part of the Japanese encephalitis serocomplex. It consists of a single-stranded positive-sense RNA genome that is contained in a virion that is approximately 50 nm in diameter [1]. The polyprotein produced from the single open reading frame is subsequently processed into ten proteins, including three structural proteins (capsid, pre-membrane/membrane, and envelope) and seven non-structural proteins (NS1, NS2A, NS2B, NS3, NS4A, NS4B, and NS5) [1]. WNV infection in vertebrates usually results in a minor or imperceptible response, although it can occasionally develop into a severe disease with central nervous system complications leading to permanent disability or death [2]. Prior to 1999, WNV was isolated to the eastern hemisphere, occurring regularly in Africa, Asia, Australia, and Europe. Since its introduction in 1999, WNV has spread throughout North, Central, and South America [3]. The epicenter of this introduction is considered the greater New York City area and it has radiated out from there. The originally introduced strain (designated NY99) was shown to be genetically similar to a strain isolated during an outbreak in Israel during 1998 [4] and was considered to be the dominant variant circulating through North America until 2002. During that year, a variant arose (designated WN02) which replaced the NY99 strain and has since become widespread throughout North America [5]. It has been suggested that the reason for this shift was due to the ability of the WN02 strain to be transmitted after two fewer days of extrinsic incubation compared to the NY99 strain, thereby giving it a competitive edge [6]. This shift occurred during 2002 and 2003, which also coincided with a peak in human cases of WNV infections [7], suggesting the importance of viral variant emergence.

As WNV has spread throughout North America, it has created occasional outbreaks corresponding to its arrival in naïve populations. The large numbers of birds affected in the initial introduction in the New York City area, in particular crows and a variety of exotic birds, were accompanied by WNV infection in humans and equines resulting in fatalities [2,4,8,9]. As WNV continued to spread throughout North America, the largest outbreak of meningitis or encephalitis ever recorded in the western hemisphere occurred in 2002 and 2003 and was directly attributed to WNV [3]. Florida, with its sub-tropical and tropical climate leading to the possibility for year-round transmission, decreased extrinsic incubation period due to increased temperatures, and transmission-competent mosquito populations alongside major bird migratory pathways and over wintering sites would appear to be fertile ground for major WNV outbreaks and diversification [10]. Conversely, there has been little WNV activity in Florida to date. The lack of WNV activity could be due to anthropogenic reasons, such as the existence of stringent mosquito control efforts already in place throughout the majority of the state, such as impoundments and aerial pesticide applications and the prevalence of climate control measures such as air conditioning and screening limiting human contact with infected mosquitoes [11]. Alternatively, this could possibly be due to the pressure of St. Louis encephalitis virus (SLEV), a native flavivirus, competing with WNV [12]. It may be that there are greater constraints on WNV movement and evolution in Florida than previously thought.

Accordingly, we undertook a genotyping study of WNV isolates from 2005 in Florida, as well as a previous isolate provided to us as a positive control, which was collected in 2003. In particular, we sequenced portions of the genome encoding the envelope protein and the NS3/NS4A region to compare our isolates to those collected throughout the country and deposited in Genbank. The region encoding the envelope protein was chosen due to its likelihood of containing antigenically relevant mutations as it is likely to undergo selection pressures due to its position on the outside of the viral capsid and subsequent interactions with host immune systems. We were also interested in determining whether or not our isolates contained a previously reported mutation in the envelope region encoding for an amino acid substitution characteristic of the WN02 strain compared to the NY99 strain, a shift from Val to Ala at amino acid 159 of the envelope region encoded for by a U to C substitution at nucleic acid position 1442. The NS3/NS4A region was chosen specifically for the high incidence of previously reported mutations in the NS3 region [13] and the importance of the NS3 region on viral replication. The NS3 region encodes for four proteins, including a serine protease involved in cleaving the translated polyprotein, as well as a nucleotide triphosphatase, a RNA 5'triphosphatase, and a helicase involved with viral RNA replication.

## Materials and methods

Mosquito pools were collected during the summer of 2005 from field sites in Duval, Indian River, and Manatee counties in Florida [14]. These field sites were selected for monitoring during the 2005 season based on WNV and SLEV activity during the preceding two years. Manatee County (27°34'25"N, 82°28'30"W), Indian River County (27°34'27"N, 80°26'11 "W), and Duval County (30°20'50 "N, 81°52'37 "W) each contained one trap site with four traps and covered a wide geographic area. The three field sites were comprised of a variety of ecosystems. Duval County is a Florida scrub ecosystem, with variety of pine trees and saw palmetto [15], while the Indian River County and Manatee County sites are both temperate hardwood forests [16]. The Indian River County site is a Sabal palm hammock located near cultivated orange and palm groves. The Manatee County site is a hardwood forest frequently inundated with standing water following a rainfall event; though it is not wet enough to be considered a hydric hammock swamp [17].

Mosquitoes were captured using lard can traps baited with a live chicken. The mosquitoes were then sorted by sex and species into pools of up to 50 after being killed en masse by freezing at -20°C. Once the pools of up to 50 mosquitoes were created, 900 μL of BA-1 diluent [18] were added to each 1.5 mL microcentrifuge tube containing the mosquitoes along with 4.5 mm zinc-plated beads (BB-caliber air gun shot). Samples were homogenized at 25 Hz for 3 min (TissueLyser; Qiagen, Inc., Valencia, CA) and centrifuged at 4°C and 3,148 × g for 4 min. The resulting mosquito homogenate was used for initial screening purposes via plaque assay and then resampled for confirmation and isolation for sequencing.

All 4009 pools created from mosquitoes collected during the 2005 surveillance period were initially screened via plaque assay and suspected positives reexamined via qRT-PCR with WNV specific primers and probe using the LightCycler^® ^480 system (Roche, Mannheim, Germany) and Superscript™ III One-Step Quantitative RT-PCR kit (Invitrogen, Carlsbad, CA). Quantitative real-time TaqMan RT-PCR was carried out as described previously for WNV [18,19]. Samples were amplified using the following operation guidelines: 48°C for 30 min, 95°C for 2 min, 45 cycles of alternating temperatures of 95°C for 10 s and 60°C for 15 s, followed by 50°C for 30 s.

Upon confirmation of the positive pools (table 1), 100 μL of the clarified supernatant from the positive mosquito pool homogenate were passed once though Vero cells. Half of the media was stored in a cryoprotective viral storage media (4% gelatin, sucrose 40%, BSA 4% in PBS pH 7.2) and frozen in vapor-phase LN_2 _and the other half of the media was considered viral stock for further testing. 250 μL of the viral stocks were neutralized and RNA was extracted according to the MagNA Pure Total NA extraction kit protocols (Roche, Mannheim, Germany).

**Table 1:** West Nile virus collection information. Positive mosquito pool numbers, locations, and dates of collection.

Isolate Number	County Collected (FL)	Date Collected
351	Duvall	23Aug2005
493	Duvall	30Aug2005
510	Duvall	30Aug2005
522	Duvall	30Aug2005
558	Manatee	1Sept2005
967	Duvall	20Sept2005
1102	Duvall	30Sept2005
2186	Indian River	1Nov2005

The subsequent RNA was eluted in a volume of 50 μL and stored at -80°C. Later, the RNA was converted to cDNA under standard thermocycling conditions using the SuperScript™ One-Step RT-PCR with Platinum^® ^Taq kit by Invitrogen (Invitrogen, Carlsbad, CA) and our specific sequencing primers (table 2). These primers were designed to amplify overlapping sections of the genome in their denoted sections. The envelope region was composed of two sections, after alignment and trimming, the completed envelope section was from nucleotide positions 1081 to 2377. The NS3 region was composed of three sections, after alignment and trimming, the completed NS3 region was from nucleotide positions 5124 to 6735.

**Table 2:** Primers.

	Forward Primer	Reverse Primer
	
Envelope 1^st^ (1042–1857)	5'-GAAGGCGATAGTTGTGTGACCA-3' (1042–1063)	5'-TGTTCCCTTCAGCTGCAACTT-3' (1834–1854)
Envelope 2^nd^ (1632–2459)	5'-CCTTGGAGCAGTGCTGGAAGTA-3' (1636–1657)	5'-TTCACGGAGAGGAAGAGCAGAA-3' (2438–2459)
NS3 1^st^ (5085–5908)	5'-CGGCTCATACATAAGCGCGAT-3' (5085–5105)	5'-TTGGTTTCACACTCTTCCGGC-3' (5888–5908)
NS3 2^nd^ (5514–6318)	5'-TTCCACAAAGGTCGAGCTAGG-3' (5514–5534)	5'-CCTAGGACCATCAAAGCACCA-3' (6298–6318)
NS3 3^rd^ (5950–6726)	5'-CCATCTGCAGTGACAGCAGCTA-3' (5950–5971)	5'-TTCGTTCCTGGAACTTCAGCC-3' (6756–6776)

Primer sequences used to sequence the envelope and NS3 regions of the samples.

*NT positions refer to amplicon location with respect to "WNV RNA, Complete Genome" GenBank accession number M12294

The resulting cDNA was dye terminated with the GenomeLab™ Dye Terminator Cycle Sequencing with Quick Start Kit (Beckman-Coulter, Fullerton, CA) and sequenced using a Beckman-Coulter CEQ8000 sequencer. The results were analyzed using the CEQ sequence analysis software to create consensus sequences which were then aligned using GeneDoc™ software to create contiguous sequences from the overlapping segments for use in phylogenetic analysis. Several phylogenetic trees were computed using MEGA 4: Molecular Evolutionary Genetics Analysis software utilizing the maximum parsimony method with 500 bootstraps along with reference sequences from GenBank (table 3). Nucleotide diversity was also calculated utilizing the MEGA 4: Molecular Evolutionary Genetics Analysis software using the maximum composite likelihood method with 1000 replicates.

**Table 3:** GenBank reference sequence information.

Abbreviation	Year of Isolation	Location	Source	GenBank accession no.
1998 Isreael	1998	Israel	*Ciconia ciconia*	AY033389
NY99	1999	Bronx Co., NY	*Phoenicopterus chilensis*	AF196835
2001 Suffolk NY	2001	Suffolk Co., NY	*Culex pipiens/restuans*	DQ164194
2002 Nassau NY	2002	Nassau Co., NY	*Culex pipiens/restuans*	DQ164195
2002 Queens NY	2002	Queens Co., NY	*Corvus brachyrhynchos*	DQ164186
2002 Indiana	2002	Indiana	Human – Plasma	DQ164200
2002 Ohio	2002	Ohio	Human – Plasma	DQ164202
2002 Georgia 1	2002	Georgia	Human – Plasma	DQ164196
2002 Georgia 2	2002	Georgia	Human – Brain	DQ164197
2002 Clinton NY	2002	Clinton Co., NY	*Corvus brachyrhynchos*	DQ164193
2002 Texas 2	2002	Texas	Human – Plasma	DQ164205
2002 Texas 1	2002	Texas	Human – Plasma	DQ164198
2002 Broome NY	2002	Broome Co., NY	*Corvus brachyrhynchos*	DQ164187
2003 Albany NY	2003	Albany Co., NY	*Corvus brachyrhynchos*	DQ164189
2003 Suffolk NY	2003	Suffolk Co., NY	*Corvus brachyrhynchos*	DQ164190
2003 Colorado 1	2003	Colorado	*Buteo jamaicensis*	DQ164204
2003 Mexico	2003	Nuevo Leon, Mexico	*Culex quinquefasciatus*	AY963775
WN-FL03p2-3	2003	Indian River Co., FL	*Culex nigripalpus*	DQ983578
2003 Colorado 2	2003	Colorado	*Pica hudsonia*	DQ164203
2003 Chautauqua NY	2003	Chautauqua Co., NY	*Corvus brachyrhynchos*	DQ164191
2003 Texas	2003	Texas	Human – Plasma	DQ164199
2003 Rockland NY	2003	Rockland Co., NY	*Corvus brachyrhynchos*	DQ164192
2003 Westchester NY	2003	Westchester Co., NY	*Corvus brachyrhynchos*	DQ164188
2004 Arizona	2004	Arizona	Human – Plasma	DQ164201
2004 Texas – Harris	2004	Harris Co., TX	*Culex quinquefasciatus*	AY712948

West Nile virus isolates used in the construction of the Envelope and NS3/NS4A phylogentic trees.

*NT positions refer to amplicon location with respect to "WNV RNA, Complete Genome" GenBank accession number

## Results

**Figure 1:**
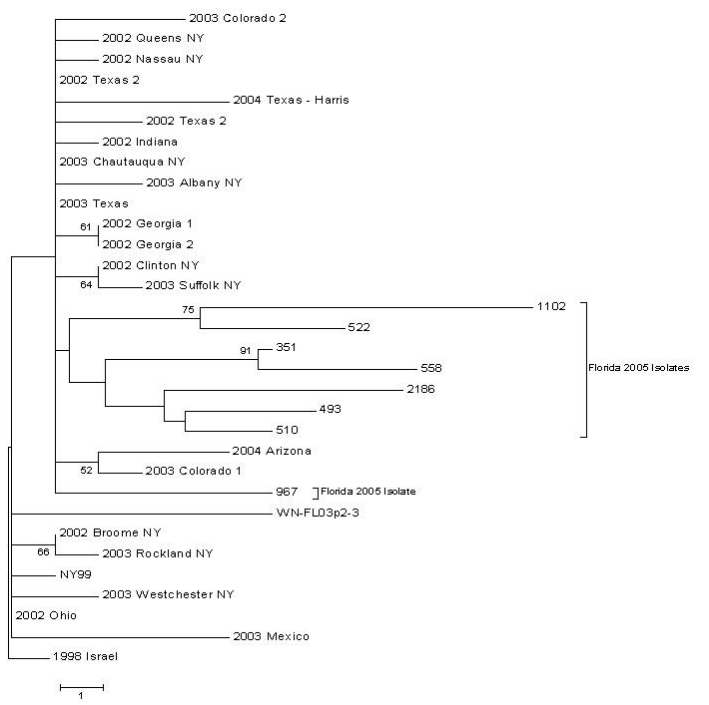
**NS3/NS4A phylogenetic tree.  **Phylogenetic tree constructed using the sequenced portion of NS3/NS4A (1611 nucleotides) of the isolates collected in Florida in 2005 and the corresponding sequences of reference files in Genbank (table 3).

Our phylogenetic analysis of the envelope sequence along with the corresponding sequences from several other strains obtained from GenBank showed that the isolates from Florida were clustered mostly together with the exception of isolate #967 (figure 1). This isolate was one of six collected over a 58 day period beginning August the 23^rd ^and ending September the 30^th^. This isolate appears to be part of the 2002 North American clade, as defined by Ebel et al. [5], yet it has two additional substitutions at nucleotide positions 2209 and 2233 (both G to A) that lead to two translated amino acid substitutions at 415 (Ala to Thr) and 423 (Asp to Asn) respectively.

**Figure 2:**
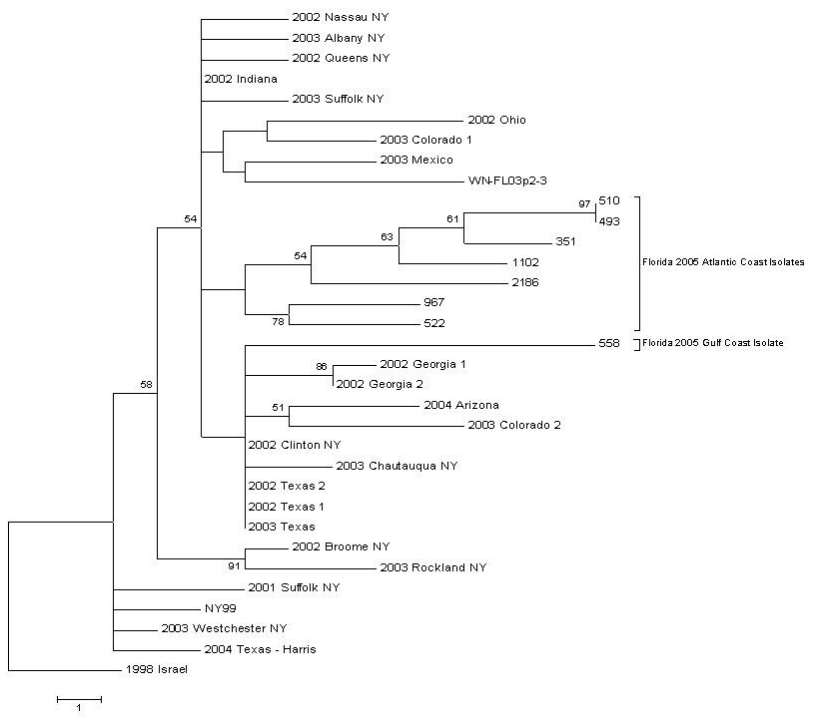
**Envelope phylogenetic tree.  **Phylogenetic tree constructed using the sequenced portion of envelope protein (1296 nucleotides) of the isolates collected in Florida in 2005 and the corresponding sequences of reference files in Genbank (table 3).

Our phylogenetic analysis of the NS3/NS4A sequences along with the corresponding sequences from several other strains obtained from GenBank showed that our isolates from Florida in 2005 grouped together, with the exception of isolate #558 (figure 2). This was the only isolate obtained from Manatee County, which is located on the Gulf (Western) coast of Florida, compared to the other sites in Duval and Indian River Counties on the Atlantic (Eastern) coast. Despite there being six positive pools collected in Duval county versus one each from Manatee and Indian River counties, minimum infection rate values, as calculated by Vitek et al., did not differ significantly geographically or temporally for this trapping period[14].

The Florida isolates contained numerous variable nucleotide and amino acid sites from the reference sequences, as well as each other; however most of these were not phylogenetically informative nor encoded any amino acid substitutions (table 4). This resulted in substantial distance calculations within the Florida 2005 isolates cohort. These distances were notably greater than the distance calculations from all other regions combined, which encompassed greater geographic and temporal domains (table 5).

**Table 4:** Nucleotide and amino acid sequence comparison of the eight WNV isolates collected in Florida during 2005.

	No. nucleotide bases			
		
Gene	Analyzed	Variable	Informative*	Mean Nucleotide Distance (%)
Envelope	1296	72	17	0.46
NS3/4A	1611	85	22	0.49

				

	No. amino acid residues			
		
Gene	Analyzed	Variable	Informative*	Mean Amino Acid Distance (%)

Envelope	432	23	7	0.50
NS3/4A	537	17	3	0.22

*Phylogenetically informative (differences occurring in two or more isolates) nucleotide and amino acid sites.

**Table 5:** Mean genetic distances between and within groups using the isolates from Florida in 2005 as a subgroup.

	Mean genetic distance* ± SE			
	
	Envelope		NS3/4A	
	
Type of comparison	NT	AA	NT	AA
Within Florida 2005	0.0086 ± 0.0016	0.0101 ± 0.0027	0.0064 ± 0.0012	0.0044 ± 0.0016
Within all other regions and times	0.0028 ± 0.0005	0.0028 ± 0.0012	0.0039 ± 0.0006	0.0015 ± 0.0005
Between Florida 2005 and all other regions and times	0.0064 ± 0.0010	0.0071 ± 0.0017	0.0061 ± 0.0009	0.0031 ± 0.0009

* = NT, nucleotide; AA, amino acid.

One silent nucleotide mutation caused the Manatee County sample 558 to cluster with several of the reference sequences that were also from the Gulf coast area or in the western part of the country. These were two samples from Georgia and three from Texas, as well as one from Arizona, one from Colorado, and two from New York from 2002 to 2004.

**Figure 3:**
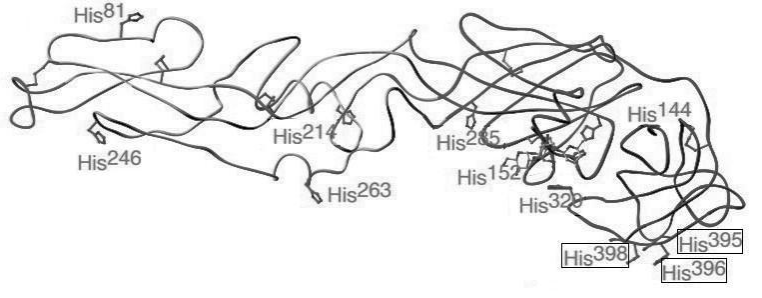
**West Nile virus envelope protein model.   **The histadines are noted and residues of interest boxed.

A cluster of amino acid substitutions was found in the envelope of sample 2186, the single isolate from Indian River County collected during 2005 during our surveillance efforts. This sample was isolated from a mosquito pool collected on November 1^st^, which makes it the last isolate collected during 2005. At three amino acid sites towards the end of the envelope protein sequence, amino acid residues 394, 397, and 400 were substituted from Asn to Ile, Trp to Gly, and Ser to Phe; respectively. These residues are associated with a set of histidines at the base of the envelope protein in domain III suggested to have a role in envelope homodimer conformation (figure 3).

## Discussion

Subsequent to our genetic analysis, it was apparent that all of our field isolates from Florida in 2005 contained the previously mentioned substitution at nucleotide position 1442 (U to C), which resulted in an amino acid substitution at E159 (Val to Ala). Interestingly, the WNV isolate collected in Indian River County during 2003 (WN-FL03p2-3) and supplied to us as our positive control did not contain that particular mutation. This leads us to believe that the timeframe for the introduction of the North American clade containing the E159 substitution into Florida was sometime between 2003 and 2005.

Furthermore, the 2005 isolates did not group with our field positive control strain FL03p2-3, which appeared more closely related to a cluster of isolates from Mexico, Colorado, and Ohio based on NS3/NS4A phylogeny. This could be the result of a difference in bird migration and overwintering patterns, such as between groups of birds flying along the eastern seaboard to overwinter in the Caribbean versus birds flying to the southeast to overwinter or continue along the Gulf coast to sites in central and South America [20].

In isolate 2186, the substitutions located immediately preceding, in between, and behind a group of histidines in Domain III of the envelope protein caught our attention. Histidines located on the envelope protein have been shown to be structurally conserved among Flaviviruses [21]. They have also been hypothesized to play a role in various conformational changes [22]. Of particular interest are the substitutions at 394 (Asp to Ile) and 400 (Ser to Phe), as these changes swap two polar residues with two nonpolar ones, perhaps leading to a change in the positioning of the neighboring histidines. Such a repositioning of these histidines could alter the conformation of the envelope, perhaps changing the antigenicity of the homodimer.

We also detected greater nucleotide diversity within the Florida 2005 isolates than within other regions as a whole, suggesting that conditions in Florida might still encourage genotypic diversification, even if transmission is low. In order to ascertain whether this was a phenomenon unique to Florida the mean genetic distances were calculated for another subset of our sequences, samples from the state of New York from 2001 to 2003 spanning comparable geographic distances but over the course of several years. Despite the larger temporal range in the samples, we found the isolates from a single year in Florida to be more diverse (table 6).

**Table 6:** Mean genetic distances between and within groups using the isolates from Florida in 2005 and New York from 2001 to 2003 as separate subgroups.

	Mean genetic distance* ± SE			
	
	Envelope		NS3/4A	
	
Type of comparison	NT	AA	NT	AA
Within New York (01–03)	0.0024 ± 0.0007	0.0031 ± 0.0015	0.0035 ± 0.0007	0.0011 ± 0.0006
Within Florida 2005	0.0086 ± 0.0015	0.0101 ± 0.0027	0.0064 ± 0.0012	0.0044 ± 0.0016
Within all other regions and times	0.0035 ± 0.0007	0.0033 ± 0.0014	0.0041 ± 0.0007	0.0017 ± 0.0007

Between New York (01–03) and Florida 2005	0.0062 ± 0.0010	0.0073 ± 0.0019	0.0059 ± 0.0009	0.0029 ± 0.0009
Between New York (01–03) and all other regions and times	0.0030 ± 0.0005	0.0032 ± 0.0013	0.0039 ± 0.0006	0.0014 ± 0.0005
Between Florida 2005 and all other regions and times	0.0067 ± 0.0011	0.0072 ± 0.0018	0.0062 ± 0.0010	0.0032 ± 0.0009

* = NT, nucleotide; AA, amino acid.

In addition, to determine if this greater nucleotide diversity within Florida during 2005 was representative of an increase in nucleotide diversity as a general trend for that year, we included representative samples from the state of Illinois collected in 2005 [23] to our mean genetic distance calculations. Within the 14 different haplotypes, as defined by Bertolotti et al., this cohort was found to have a mean genetic distance of 0.0040 ± 0.0007, based upon samples obtained from GenBank and aligned with our Florida 2005 isolates. In comparison, the cohort of isolates collected in Florida during 2005 had a mean genetic distance of 0.0089 ± 0.0015, suggesting differing evolutionary constraints between these two regions.

## Conclusion

Taken together, these findings expand our understanding of the temporal and spatial compartmentalization of West Nile virus subtypes within North America. It would appear that the introduction of the North American clade into Florida occurred sometime between 2003 and 2005. The method of introduction may have a geographic or migratory component, due to a similarity of the original isolate to those from Ohio, Colorado, and Mexico that is not seen with the isolates from 2005. Along with the greater genetic diversity among the isolates collected in 2005 compared to those from larger geographic and temporal zones, an isolate was collected that contained several amino acid substitutions associated with histadine residues located in biologically important areas of the envelope protein. These findings confirm the need to continue to monitor and highlight the uniqueness of the development of the West Nile virus in Florida.

## Competing interests

The authors declare that they have no competing interests.

## Authors' contributions

DC isolated and sequenced the viruses from the positive pools, constructed the consensus sequences and aligned them, performed the phylogenetic analysis, and contributed to the writing of the manuscript. CM was the overall project coordinator and contributed to experimental design, data analysis, and writing of the manuscript.
